# Detection of Khapra Beetle Environmental DNA Using Portable Technologies in Australian Biosecurity

**DOI:** 10.3389/finsc.2022.795379

**Published:** 2022-02-11

**Authors:** Alejandro Trujillo-González, David N. Thuo, Uday Divi, Kate Sparks, Thomas Wallenius, Dianne Gleeson

**Affiliations:** ^1^EcoDNA Group, Centre for Conservation Ecology and Genomics, University of Canberra, Canberra, ACT, Australia; ^2^Biosecurity Strategy and Reform Division, Department of Agriculture, Water, and the Environment, Canberra, ACT, Australia; ^3^Science and Surveillance Group, Biosecurity Plant Division, Department of Agriculture, Water and the Environment, Canberra, ACT, Australia

**Keywords:** *Trogoderma granarium*, surveillance, border control, eDNA, biosecurity

## Abstract

Khapra beetle, *Trogoderma granarium* Everts, 1898, is a serious pest of stored grain products globally. Environmental DNA (eDNA)-based methods offer sensitive detection tools used to inform biosecurity officers on the presence of high-risk pests. This study tested laboratory and portable molecular technologies to detect khapra beetle environmental DNA extracted from dust samples collected during biosecurity responses (Tuggeranong and Fyshwick) to khapra beetle incursions in Australia. Airborne and floor dust samples were collected opportunistically using handheld vacuum cleaners and eDNA was extracted using either field or laboratory-based extraction methods and analyzed using laboratory benchtop real time PCR machines and portable machines with two TaqMan and one LAMP-based assay. We successfully collected, extracted, and amplified khapra beetle eDNA from dust samples by qPCR, but failed to amplify *T. granarium* eDNA using LAMP. The Laboratory qPCR machine showed significantly higher mean Ct values (*p* < 0.001) and significantly higher positive detections for both assays (*p* < 0.001) compared to the portable thermocycler. DNA yield was significantly higher in samples extracted using laboratory-based kits compared to field kits (*p* < 0.001) for both vacuumed and airborne samples (Mean DNA ± S.D. = 5.52 ± 4.45 and 4.77 ± 1.68 ng/μL, respectively), compared to field kits, (1.75 ± 1.17 and 1.36± 1.29 ng/μL for vacuumed and airborne samples, respectively). There were no significant differences in DNA yield between collection methods or differences in amplification associated to extraction or collection methods in either platform tested in this study. Portable technologies tested in this study (Franklin™ Real Time Thermocycler and Genie III) accurately amplified all tissue derived DNA during assay optimisation and field testing, highlighting the capacity of these technologies to complement biosecurity in confirming specimen ID. There was a high incidence of positive detections in field negative controls (Tuggeranong = 12.3 % and Fyshwick = 50 %), mostly attributed to the use of contaminated vacuum cleaners. We discuss suitable methods to minimize sample cross-contamination, the potential of portable molecular technologies as tools for biosecurity applications, and the suitability of eDNA-based molecular detection methods to complement global trade biosecurity for one of the most invasive and important grain pests worldwide.

## Introduction

Khapra beetle, *Trogoderma granarium* Everts, 1898, is among the most damaging insect pests of stored grain products. This beetle has a highly resistant larval stage that can survive at a wide range of abiotic conditions (1, 2), a generalist feeding habit affecting the most important grains in the global trade (2–5), the ability to infest non-grain products such as spices, nuts, oilseeds, dried fruits or dried vegetables (3, 6) and a capacity to survive in small crevices and other small refugia (2, 7). Khapra beetle is exotic to Australia but is currently established in ~83 countries throughout Asia, Africa, the Middle East, and Europe (8) and has been globally identified as one of the most destructive insects of stored grains and foodstuff (2, 7, 9).

Khapra beetle is currently considered the most important national priority plant invertebrate pest and the most important plant priority pest for the grains industry in Australia (10). Although this species is not established within Australia, it is a highly invasive quarantine pest and a widespread incursion could cost the country $15.5 billion over 20 years. As such, the Australian government, through the Australian Department of Agriculture, Water and the Environment (DAWE) initiated an urgent action plan in 2020 that includes prevention of entry of high-risk plant products within unaccompanied and accompanied baggage or via international travelers or mail articles (11), and mandatory treatment of certain high risk shipping containers (12).

Khapra beetle has been detected as a contaminant pest in imported goods such as furniture, household appliances, associated packaging, and shipping containers. For example, in August and November of 2020, DAWE was notified of the presence of unusual beetle larvae infesting packaged household items purchased from retail stores in the Australian Capital Territory (ACT) and New South Wales (NSW). These detections triggered biosecurity responses, which involved biosecurity officers visually inspecting the area for obvious pest detections, followed by vacuuming and sweeping dust samples for detailed examination by trained entomologists. Entomologists then sieved and exhaustively examined each sample under a dissecting microscope for *T. granarium* or *Trogoderma*-like larvae, adults, or fragments, which were confirmed using morphological (13) and molecular diagnostic protocols (14). Such responses require substantial resources and time to accurately process all dust samples and avoid potential false negative outcomes. As such, there is a need to test and develop molecular methods that complement the needs of biosecurity officers during biosecurity responses. Environmental DNA (eDNA)-based methods have been shown to be cost-effective reliable methods to inform users on the presence of target species in surveillance applications (15), with studies showing how eDNA-based detection using soil and airborne dust samples can provide valuable data on species presence and diversity (16–18). When used with portable, point-of-care technologies, eDNA-based detection can offer sensitive detection tools to support management of biosecurity risks (19), ADDIN EN.CITE (16–18)but no studies have so far tested suitable extraction methods of dust samples and the utility of portable technologies during biosecurity applications.

The aim of this study was to determine if *T. granarium* eDNA could be extracted from dust samples and detected using molecular methods during onshore biosecurity responses. This study tested portable molecular technologies to detect khapra beetle environmental DNA during biosecurity responses using published molecular assays designed to identify *T. granarium* (14, 20, 21) and highlights multiple obstacles when using field-based molecular tools during biosecurity responses. We discuss the potential of portable molecular technologies as viable tools for biosecurity officers in Australia and the suitability of eDNA-based methods to complement global trade biosecurity for one of the most invasive and important grain pests worldwide.

## Methods

### Environmental DNA Sample Collection

Environmental DNA sampling at confirmed *T. granarium* detection sites was subject to government approval and access, occurring where biosecurity response measures had already been initiated by DAWE under the Emergency Plant Pest Response Deed. As such, testing of eDNA collection methods and portable technologies was opportunistic, and subject to available timeframes within which approval was given to sample each location. Samples were collected from two separate ACT retail stores that had received and stored goods infested with khapra beetle in August (Tuggeranong, Canberra) and November (Fyshwick, Canberra) of 2020. Both biosecurity responses were triggered by private citizens reporting unusual beetle larvae crawling inside purchased household items. In both instances, *T. granarium* specimens were recovered by biosecurity officers inside the packaging and within the cardboard layers of the boxes where each purchased item was kept. Specimens were morphologically identified by DAWE entomologists following the international standard for *T. granarium* as outlined by the International Plant Protection Convention (13), and by molecular identification using a Loop-mediated isothermal amplification (LAMP) assay (14) and confirmed by Sanger sequencing. Environmental DNA sampling was undertaken during biosecurity delimitation responses at each site following detection of *T. granarium*.

#### Tuggeranong Detection Event-August 2020

Biosecurity officers directed retail outlet staff to clear a 5 m radius area from where the infested refrigerators were kept prior to purchase. Officers then partitioned the area into six 3 × 3 m quadrants, forming a grid, which allowed for methodical vacuum sampling of the adjacent potentially infested areas. Environmental DNA samples were collected simultaneously using two methods. In the first method, officers vacuumed each grid using four vacuum cleaners; three handheld (Black & Decker PET Dustbuster) and one commercial vacuum cleaner provided by the retail store (brand unknown). Vacuum cleaners were used randomly to vacuum each grid by biosecurity officers for ~4 min. The content of each vacuum cleaner was then emptied inside a plastic bag and labeled for further visual inspection by entomologists of DAWE. Vacuum cleaners were not sterilized between grids and cross-contamination could not be prevented. After each grid was vacuumed, six dust and debris samples (~10 g per sample; herein referred to as dust samples) were collected using sterile single use forceps from inside each plastic bag labeled by biosecurity officers. Three samples were placed directly inside 5 mL tubes with 2.5 mm ball bearings and 3 mL of Biomeme Lysis Buffer (Biomeme, Inc.) and the remaining three samples were placed inside 5 mL tubes with 4 mL of 80% ethanol.

The second method involved the use of a separate handheld vacuum cleaner (Dyson V7 cord-free vacuum) attached to a plastic filter casing with a 42 mm, 1.2 μm pore size cellulose nitrate filter paper (Sterlitech, Inc.) to sample airborne dust. This method was based on a previously tested protocol used to sample for dust samples in field conditions (Gleeson, *pers. Comm*.). This vacuum cleaner had not been previously used by biosecurity officers and was sterilized with 2.5% bleach before the sampling event. Airborne dust within each grid was vacuumed for ~15 s, ~30 cm above the area of each quadrant. Filters were then carefully removed from the casing using sterile single-use forceps and placed inside 5 mL tubes with 2.5 mm ball bearings containing either 3 mL of Biomeme Lysis Buffer (Biomeme, Inc.) or 4 mL of 80% ethanol. The filter casing and vacuum cleaners were then wiped clean using paper towels and 1% bleach. A total of six airborne samples were planned to be collected from each quadrant, however, time limitations allowed for two filters to be collected from 3 out of six quadrants. All samples were immediately taken to the University of Canberra for eDNA extraction.

A total of eight field negative controls were collected. Three were 5 mL tubes 3 mL of Biomeme Lysis Buffer (Biomeme, Inc.), three 5 mL tubes with 4 mL of 80% ethanol and two consisted of filter papers placed inside the plastic filter casing attached to the handheld vacuum cleaner, placed inside either a 5 mL tube with 3 mL of Biomeme Lysis Buffer or 4 mL of 80% ethanol. Field controls were collected by opening each tube and walking across all quadrants while officers were vacuuming. Following filter paper controls were collected by vacuuming the air at eye-level (~185 cm) while walking across all quadrants while officers were vacuuming.

#### Fyshwick Detection Event-November 2020

Retail outlet staff had vacuumed the area prior to our arrival following instructions by DAWE and had already cleared a 5 m radius area from where the infested baby highchair was kept prior to purchase. Given that the area had already been vacuumed by retail staff, no grids were used during this biosecurity response. In this occasion, officers vacuumed the whole 5 m area using a large commercial dry vacuum cleaner with internal single-use paper filter bags (VAX®, Australia) for ~15 min. This vacuum cleaner had been used during a separate *T. granarium* detection event 3 days prior, and it was unclear if officers used single-use filters during that event. For this reason, two dust samples were taken from the inside filter of the vacuum cleaner using sterile, single use forceps to determine potential cross-contamination and placed directly inside a 5 mL tube with 2.5 mm ball bearings and 3 mL of Biomeme Lysis Buffer (Biomeme, Inc.). After vacuuming, eight dust samples (~10 g of sample) were collected from the paper filter of the vacuum cleaner using sterile single use forceps and placed directly inside 5 mL tubes with 2.5 mm ball bearings and 4 mL of Biomeme Lysis Buffer (Biomeme, Inc.).

Environmental DNA samples were processed and analyzed at the site to test operational use requirements during this biosecurity response. All samples were processed using the M1 Bulk Sample Prep Kit for DNA-HI (Biomeme Inc.) on-site. Given indications that there was potential for khapra beetle eDNA contamination from a prior biosecurity response, the two samples collected from the vacuum cleaner were tested before any others using two Franklin™ Real Time Thermocyclers. Both samples were run in triplicate in each thermocycler with a separate strip containing two non-template controls and one genomic positive control. Each thermocycler ran one of the two *T. granarium* TaqMan assays tested in this study.

### Confirmed Positive EDNA Sample and Positive Controls

A separate vacuumed sample was provided by DAWE collected from the car boot of an individual who purchased a refrigerator infested with *T. granarium* during a third detection event in the suburb of Kambah (Canberra, ACT, Australia). This sample was collected by biosecurity officers by sweeping/vacuuming using a handheld vacuum cleaner and confirmed contain a live *T. granarium* larva. The larva was sent for molecular identification, while the dust sample was kept at room temperature inside a plastic zip-lock bag at the Commonwealth Scientific and Industrial Research Organization (CSIRO) Black Mountain site in Canberra for ~5 days prior to being delivered to the University of Canberra for DNA extraction.

Biosecurity officers also provided 10 separate vials with *T. granarium* larvae and adult specimens collected during the initial detection at the Tuggeranong site as positive controls for testing during qPCR amplification. Genomic DNA from each vial was extracted using a DNeasy Blood and Tissue Kit (Qiagen) following the manufacturer's instructions. All samples were stored at −20°C.

### Environmental DNA Extraction

All samples kept in Biomeme lysis buffer collected during the Tuggeranong and Fyshwick detection events were extracted using M1 Bulk Sample Prep Kits for DNA-HI (Biomeme Inc.). Two extraction negative controls (5 mL tubes with 3 mL of Biomeme lysis buffer) were prepared and processed together with samples to assess extraction level cross-contamination. Each tube was shaken vigorously for 2 min and 1 mL of lysis buffer from each tube was collected sequentially using a Biomeme syringe filter attached to sterile 1 mL syringe and processed following the manufacturer's protocol. Each sample was eluted in 500 μL of Biomeme Elution buffer and stored at −20°C.

All samples kept in 80% ethanol and the confirmed positive sample from Kambah were processed using a DNeasy Blood & Tissue Kit (Qiagen). Two extraction negative controls (1.7 mL Eppendorf tubes with 180 μL of lysis buffer and 20 μL of Proteinase K) were prepared and processed together with samples to assess extraction level cross-contamination. Dust samples from each tube were placed on a sterile glass surface and a small fraction (~5 g) was placed inside a 1.7 mL Eppendorf tube with 180 μL of lysis buffer and 20 μL of Proteinase K. Tubes were then placed on a rocker and incubated at 56°C inside a hybridizing oven for 1 h. Samples were then processed following the manufacturer's protocol and eDNA eluted in 50 μL of MilliQ water. A total of 15 replicate samples were extracted from the Kambah dust sample given its importance for assay testing and optimisation. DNA yield of each extract was measured using a Thermo Scientific™ NanoDrop™ One Microvolume UV-Vis Spectrophotometer and then stored at −80°C.

### Trogoderma Granarium TaqMan Assay Optimisation

Samples collected in this study were tested for the presence of *T. granarium* eDNA using two published probe-based assays (20, 21) (Table 1). The Olson assay targeted a 248 bp fragment of the 16s gene region (21), while the Furui assay targeted an 83 bp fragment of the ND6 (NADH dehydrogenase VI region) (20). Both assays were optimized for the purpose of testing environmental samples for the presence of *T. granarium* DNA following minimum quality standards for qPCR testing (22). Both assays were *in silico* tested for specificity using the BLAST search function on the National Centre for Biotechnology Information (NCBI) website. The Olson assay has been validated as a diagnostic assay to confirm the identity of *T. granarium* specimens targeting tissue-derived DNA and has undergone specificity testing against co-occurring and phylogenetically related dermestid species in Australia (*National Diagnostic Protocol, under review*). The Furui assay has not been formally validated as a diagnostic assay but was tested for sensitivity and specificity by the authors (20). This study complements prior specificity testing of each assay by testing both assays against two separate specimens of *Trogoderma variabile* Ballion, 1878, and native *Trogoderma, Anthrenus, Anthrenocerus, Attagenus* and *Orphinus* (Coleoptera: Dermestidae) tissue samples provided by the Science & Surveillance Group from DAWE.

**Table 1:** TaqMan and Loop-mediated Isothermal Amplification assays used to amplify *Trogoderma granarium* eDNA.

**Assay**	**Primer**	**5^**′**^-Sequence-3^**′**^**	**Size (bp)**	**Reference**
Modified Olson assay	der16SF4	CTAAAATTGAAAATTTCTATACT	248	Olson et al. (21)^*^
	der16SR1	CTAGCCTGCTCCCTGATTGA		
	P1	FAM- TGACTGTGCGAAGGTAGCAT-QSY		
Furui assay	Furui F	CAGCCTTATATGACTTCTCATACC	83	Furui et al. (20)
	Furui R	GATTTCATGTTGGGAATGATG		
	Furui P	FAM- GCAAATGGTGGCGAGTGTTGTC-QSY		
LAMP assay	Khapra_F3	GGTAATTTAATCTTATAATCACAAGATGG	234	Rako et al. (14)
	Khapra_B3	AACTGGAATGAATGGTTGGACGAA		
	Khapra_FIP	TTGTTAGTATAGAAATTTTCAATTTTAGGATCATCTAATCATAAATCAATGTTTCA		
	Khapra_BIP	TTTAACAATTAAAGAAATAATAAAACTCTTGATTACTGTCTCTTTTTTATTTTG		
	Khapra_Floop	TTAATTTGGTTGGGGTGACTA		
	Khapra_Bloop	CGTCTTTTAAAAAAATTTGAGCC		

* *The probe for this assay was modified from its original sequence (21)*.

The analytical sensitivity of each TaqMan assay was assessed by obtaining the limit of detection (LOD) using eDNA and synthetic standards designed for each assay (Supplementary 1). Standard curves were established using dilution series of known concentrations ranging from 10^7^ copies/μL and decreasing tenfold down to 1 copy/μL. The same was done with eDNA extracted from the confirmed positive dust sample collected in Kambah, from starting concentration of 10 ng/μL down to 10^−6^ ng/μL. Six PCR replicates were used in each dilution step to assess LOD. The LOD was assessed as the last dilution of the standard curve wherein the targeted DNA amplified in all qPCR technical replicates (23). Given that at the time of testing there was one confirmed positive eDNA sample collected in Kambah (Canberra, Australia), this study provides a preliminary evaluation of accuracy by examining positive amplification of qPCR technical replicates.

Each TaqMan assay was optimized for use in a ViiA™ 7 Real-Time PCR System (Applied Biosystems, Australia) and in a Franklin™ Real Time Thermocycler (Biomeme Inc., USA) to test eDNA. Each reaction in the ViiA™ 7 Real-Time PCR System consisted of 10 μL TaqMan Environmental Master Mix 2.0 (ThermoFisher), 1 μL of each of primer and probe (10 μM) for the Olson assay or 0.8 μL (10 μM) for the Furui assay, 5 μL of template, and PCR water for a total volume of 20 μL. Reactions in the Franklin™ Real Time thermocycler consisted of 10 μL of 2× LyoDNA 2.0 + IPC Master mix (Biomeme, Inc.), 1 μL of each of primer and probe (10 μM) for the Olson assay or 0.8 μL (10 μM) for the Furui assay, 5 μL of template and PCR water for a total volume of 20 μL (Supplementary 2).

Cycling conditions differed between platforms. In the ViiA™ 7 Real-Time PCR System, conditions for the Olson assay were: 95°C (10 min), followed by 50 cycles of 95°C (20 s), 50°C (1 min) and 72°C (30 s) ramping at 2.42°C/s, followed by a final holding stage at 4°C. Conditions for the Furui assay were: 95°C (10 min), followed by 45 cycles of 95°C (20 s) and 60°C (20 s) ramping at 2.42°C/s, followed by a final holding stage at 4°C. In the Franklin™ Real Time Thermocycler, conditions for both assays were optimized for field-based operational use. Cycling conditions for the Olson assay were optimized as follows: 95°C (2 min), followed by 50 cycles of 95°C (10 s) and 50°C (30 s). Conditions for the Furui assay were 95°C (2 min), followed by 45 cycles of 95°C (20 s) and 60°C (10 s). Samples in both platforms were run in triplicate with positive and non-template controls. All positive controls in this study were genomic DNA extracted from confirmed *T. granarium* larvae or adults collected by biosecurity officers during each event.

### Trogoderma Granarium LAMP Assay Optimisation

A LAMP assay designed to detect a 234 bp fragment in the 16S gene region of *T. granarium* was tested against eDNA (14) (Table 1). This assay was designed as a rapid molecular method to identify *T. granarium* specimens. The assay has been tested for specificity against 23 non-target Dermestidae species, showing no non-target amplification, and was validated to detect as little as 1.0^−6^ ng/μL of *T. granarium* DNA extracted from larvae and adult specimens (14). This assay is currently accepted by DAWE to confirm the identity of *T. granarium* specimens collected during biosecurity responses in laboratory conditions (14). This assay was tested in laboratory conditions against confirmed positive eDNA extracts from samples collected in Kambah. Reactions were undertaken in a handheld Genie III machine (OptiGene, UK) at the University of Canberra. Each reaction consisted of 14 μL Isothermal Master Mix Iso-001 (Geneworks, Australia), 10 μL of primer mix (14) and 1 μL of eDNA or DNA template for a total volume of 25 μL. Each run in the Genie III consisted of 6 technical replicates of the eDNA positive sample, one positive DNA controls and one non-template control. Isothermal amplifications conditions were 65°C for 25 min followed by a ramping step from 98 to 73°C at 0.05°C/s.

### Real Time PCR Positive/Negative Determination and Statistical Analyses

PCR replicates for the eDNA samples were putatively positive if amplification curves crossed a common fluorescence threshold determined by the inclusion of positive controls within each qPCR run. Replicates where no amplification was observed above a common threshold were deemed negative. Putative positive amplicons and replicates with amplification beyond each assay's limit of detection were purified using a PCR purification Kit (Qiagen) following the manufacturer's instructions and sent for Sanger sequencing to the Biomolecular Resource Facility (BRF) at the John Curtin School of Medical Research at the Australian National University (JCSMR, ANU) for species amplification confirmation. Mean DNA yields were compared between sites and methods using non-parametric Mann-Whitney U tests as data was not normally distributed, and mean cycle thresholds (Ct) of confirmed positive detections were compared between sites, assays and collection methods using two-way ANOVAs followed by Tukey's HSD tests (SPSS Statistics 23.0.0).

## Results

### Environmental DNA Extraction Yield and Amplification

**Figure 1:**
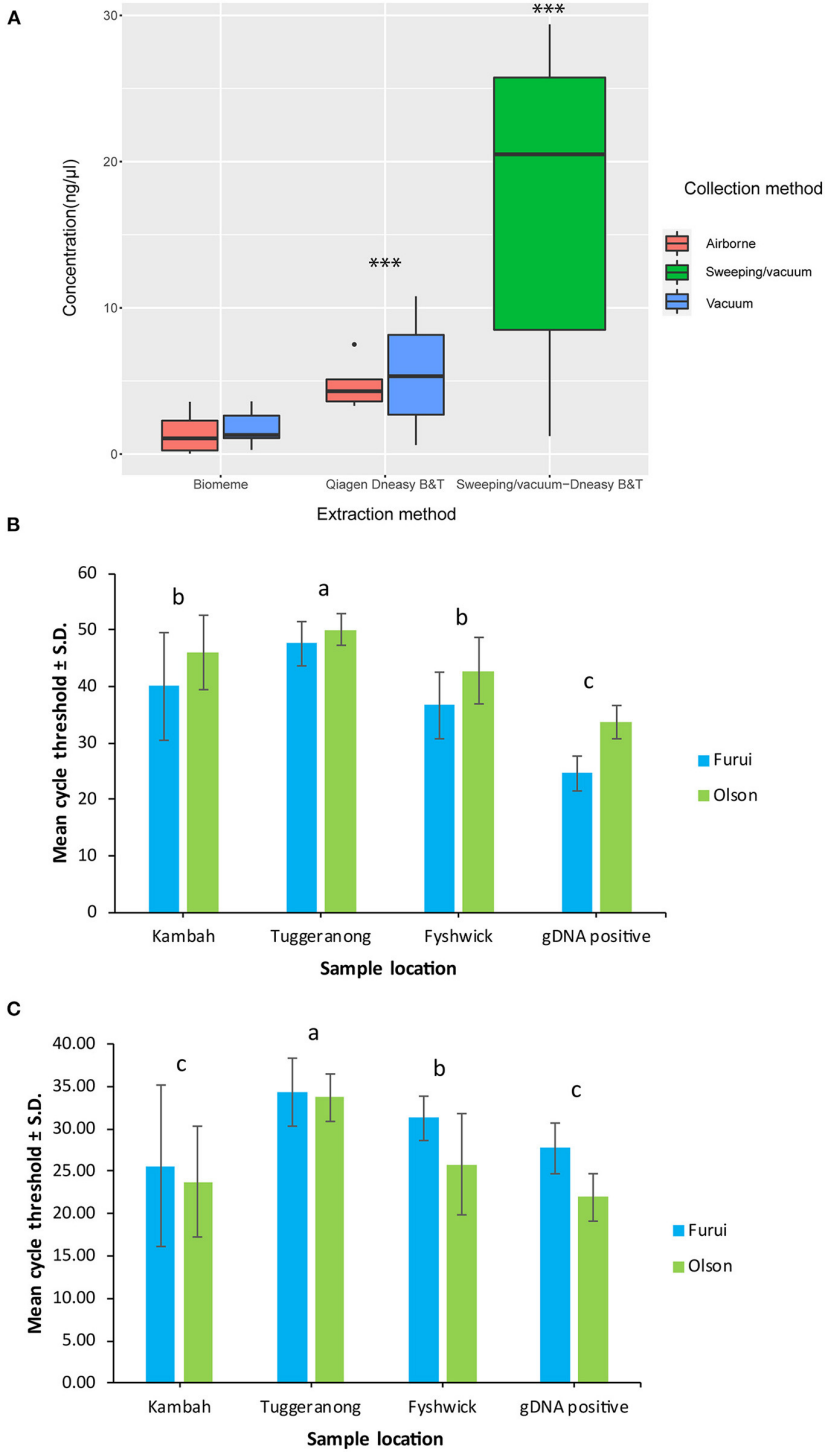
Mean environmental DNA yield achieved by either Biomeme or Qiagen Dneasy Blood & Tissue kit extraction protocols from dust samples collected during biosecurity responses in Tuggeranong (vacuum) and Kambah (sweeping/vacuum) **(A)** and mean cycle threshold values of environmental DNA and genomic DNA samples collected during biosecurity responses in Kambah, Tuggeranong and Fyshwick achieved with the Olson and Furui assays in the ViiA™ 7 Real-Time PCR System **(B)** and in the Franklin™ Real Time thermocycler **(C)**. ***Statistical differences using non-parametric Mann-Whitney U tests. ‘a’ and ‘b’ and ‘c’ indicate differences between pairs of means determined using Tukey's HSD tests of statistically significant two-way ANOVAs.

There was a significant difference in mean DNA yield achieved between extraction methods used to extract DNA from vacuum and filter samples collected in Tuggeranong. Yields were significantly higher using the Qiagen Dneasy Blood and Tissue Kits with means of 5.52 ± 4.45 ng/μL in vacuum samples and 4.77 ± 1.68 ng/μL in airborne samples, compared to the Biomeme protocol of 1.75 ± 1.17 ng/μL in vacuum samples and 1.36 ± 1.29 ng/μL in airborne samples (Mann-Whitney U Test, *p* < 0.001) (Figure 1A). There were no significant differences in DNA yield between collection methods within each extraction method (Figure 1A). In comparison to the dust eDNA samples collected in Tuggeranong, mean DNA yield from the Kambah sample was 17.27 ± 11.61 ng/μL (*n* = 15 technical replicates). There was no amplification of extraction negative controls.

The ViiA™ 7 Real-Time PCR System showed significantly higher mean Ct values [two-way ANOVA, *F*_(1,371)_ = 197.557, *p* < 0.001] and significantly higher positive detections for both assays [two-way ANOVA, *F*_(1,371)_ = 9.172, *p* < 0.001] compared to the Franklin™ Real Time thermocycler. There were no significant differences associated to extraction or collection methods in either platform tested in this study (Figure 1).

Amplification of *T. granarium* eDNA in the ViiA™ 7 Real-Time PCR System showed significant differences between sampling locations [two-way ANOVA, *F*_(3,179)_ = 45.910, *p* < 0.001] and assays [two-way ANOVA, *F*_(1,179)_ = 12.051, *p* < 0.001]. Samples from Tuggeranong displayed significantly higher Ct values compared to all other sites, with mean Ct ± S.D. of 47.62 ± 4.01 and 50.05 ± 2.80 with the Furui and Olson assay, respectively (Figure 1B). Replicates from the Kambah sample showed mean Ct ± S.D. of 40.03 ± 9.5 and 46.01 ± 6.59 with the Furui and Olson assay, respectively (Figure 1B), while samples collected in Fyshwick showed 36.674 ± 5.85 Ct with the Furui assay and 42.75 ± 5.95 Ct with the Olson assay (Figure 1B). there were no significant differences in mean Ct of samples collected in Kambah and Fyshwick. However, it is important to note that samples from Fyshwick were collected with a contaminated vacuum cleaner used during a separate biosecurity response of khapra beetle.

Similarly, amplification of *T. granarium* eDNA in the Franklin™ Real Time thermocycler showed significant differences between locations [two-way ANOVA, *F*_(1,100)_ = 12.090, *p* < 0.001], where Samples from Tuggeranong displayed significantly higher Ct values than all other sites, with mean Ct ± S.D. of 34.87 ± 2.88 and 33.69 ± 2.40 with the Furui and Olson assay, respectively (Figure 1C). Samples collected in Fyshwick showed 31.71 ± 4.64 Ct with the Furui assay and 25.76 ± 1.97 Ct with the Olson assay (Figure 1C). Lastly, technical replicates from the Kambah sample showed mean Ct ± S.D. of 25.60 ± 5.94 and 23.74 ± 5.26 with the Furui and Olson assay, respectively (Figure 1C). There were no significant differences between in mean Ct values between assays used in the Franklin™ Real Time thermocycler.

Field negative controls collected in both biosecurity responses showed positive amplification of *T. granarium* eDNA using both assays in both the ViiA™ 7 Real-Time PCR System and the Franklin™ Real Time thermocycler. Specifically, 12.3% and 50% of qPCR technical replicates from field negative controls collected from Tuggeranong and Fyshwick (Total number of technical qPCR replicates/total number of positive technical qPCR replicates = 8/65 and 6/12, respectively, Table 2), respectively, were confirmed positive for *T. granarium* eDNA.

**Table 2:** Number of confirmed positive technical qPCR replicates for *Trogoderma granarium* environmental DNA from samples and field negative controls collected during biosecurity responses in Fyshwick and Tuggeranong.

**Sample type**	**Location**	**assay**	**Total technical qPCR replicates**	**Positive qPCR replicates**
Dust sample	Fyshwick	Furui	26	24
		Olson	24	14
	Tuggeranong	Furui	221	105
		Olson	226	31
Field control	Fyshwick	Furui	6	5
		Olson	6	1
	Tuggeranong	Furui	29	7
		Olson	36	1

### TaqMan Assay Optimisation and Reproducibility

Both TaqMan assays successfully amplified *T. granarium* eDNA extracted from dust samples in laboratory and field conditions. The Olson assay had an LOD of 100 copies/μL (mean C_T_ ± SD = 48.17 ± 1.44) with an *R*^2^ = 0.99 and efficiency = 93%. Similarly, the LOD for the Furui assay was estimated to be 10 copies/μL (mean C_T_ ± SD = 40.78± 0.44) an *R*^2^ = 0.96 and efficiency = 91%. Positive controls amplified in all plates and no amplification occurred in the negative template controls. All positive detections of eDNA and genomic DNA samples were confirmed by Sanger sequencing to display 98.7–100% similarity with *T. granarium* sequences in NCBI (NCBI no. MT113335) and a selected number of sequences were accessioned for future studies (NCBI no. MW911673-MW911691). Positive amplicons obtained using the Furui et al. (20) assay that amplified within assay cut-off values consistently showed poor sequencing quality due to the small size of the fragments, while all positive amplicons outside cut-off values were confirmed to be amplification errors. There was no amplification of any of the provided tissue samples from non-target Australian native specimens with either of the tested assays.

TaqMan assays successfully amplified khapra beetle eDNA in the confirmed positive eDNA sample provided to this study from a private residence in Kambah (Australia). Assay reproducibility for the Olson assay was 66% (positive technical replicates/total technical replicates = 18/27) and 100% (positive technical replicates/total technical replicates = 9/9) in ViiA™ 7 Realtime PCR system and Franklin™ Realtime Thermocycler, respectively. In the same way, the Furui assay had a detection success rate of 90% (positive technical replicates/total technical replicates = 29/32) and 100% (positive technical replicates/total technical replicates = 6/6) in the ViiA™ 7 Realtime PCR system and Franklin™ Realtime Thermocycler, respectively. The LAMP assay failed to detect khapra beetle eDNA in the confirmed eDNA sample provided to this study (positive technical replicates/total technical replicates = 0/24) and was not selected for further testing. Environmental DNA extracts from this confirmed positive eDNA sample were heavily used during assay optimisation, resulting in differences in available technical replicates to assess assay reproducibility in the portable Franklin™ Thermocycler.

The Franklin™ Real Time Thermocycler accurately amplified all tissue derived positive control DNA used during assay optimisation and field testing of the technology using the Olson assay. Similarly, the LAMP assay tested in this study using a Genie III accurately amplified all DNA positive controls must faster than the qPCR-based assays tested in this study (detection <25 min), highlighting the capacity of these technologies to complement biosecurity requirements in confirming specimen ID.

## Discussion

The use of eDNA-based molecular techniques for exotic species identification are increasingly promising tools to inform biosecurity (24). For these methods to be adopted, the sampling, processing, and analyzing samples must be technically feasible, precise, and repeatable (24). Surveillance applications using eDNA-based detection have been demonstrated to reliably inform end users on the presence and activity of exotic species to better manage biosecurity risks and improve detection probability (15, 24–26). Border control and onshore biosecurity responses stand out as unique applications due to their importance in trade and legislation. These applications require standard protocols and methods to inform decisions that must be held to scrutiny in a governmental level (27). In order to reach such certainty, research must explore operational requirements and limitations of molecular technologies to better manage sources of error (28). This study presents results from Australian border biosecurity responses to the detection of *T. granarium* specimens contaminating non-agricultural commodities imported from overseas. We showed that khapra beetle eDNA can be extracted, and amplified from dust (vacuumed and airborne) samples and tested using qPCR TaqMan assays. Lastly, we highlight a substantial proliferation of false positive results associated to cross-contamination during sample collection that must be addressed for eDNA-based methods to be implemented in government biosecurity responses and applications.

Sample collection methods used in this study were prone to cross-contamination during sample collection within (Tuggeranong) and between (Fyshwick) sampled sites. False positive results were attributed to the opportunistic nature of sample collection, as biosecurity officers were not informed beforehand of the requirements needed to minimize cross-contamination. Officers carried out each biosecurity response as per guidelines and legislation approved by DAWE, which do not outline requirements for eDNA testing at these early stages of implementation. As such, the priority at each site was to detect and remove *T. granarium*, followed by cleaning of all contaminated surfaces and fumigation. Within this context, the effect of false positive detections due to the use of contaminated vacuum cleaners had no impact on the outcome of each response as officers had confirmed the presence of *T. granarium*, nonetheless, such an obstacle highlighted the need to minimize eDNA cross-contamination in biosecurity. In the absence of suitable measures to minimize false positive and negative errors, eDNA-based testing could become an unreliable tool to inform officers on the presence of high priority pests during border control and onshore applications, which would have important trade implications if the technology was used to assess trade compliance and regulations. Future studies should consider the use of equipment that can be easily attached to handheld vacuum cleaners to collect and isolate dust samples (e.g., dust interceptors) and use rigorous cleaning protocols to reduce the sample cross-contamination. Risk management measures must also be implemented to manage the possibility of false positive and negative errors associated to field-based eDNA testing, which include sample collection for lab-based testing, using multiple rather than single assays to test samples, and amplicon identity confirmation by sequencing.

Further testing is required to better gauge assay reproducibility with both TaqMan assays tested in this study and better assess the capacity of LAMP to amplify insect eDNA in dust samples. There was only one sample from which detection success could be assessed appropriately for the purpose of eDNA testing in this biosecurity setting, which was also used for assay optimisation and assessing analytical sensitivity. As such, both TaqMan and LAMP assays were tested against a single environmental matrix for the purpose of assessing assay reproducibility. Real Time PCR has been routinely used to detect eDNA from a broad range of surveillance applications (15) and was shown by this study to successfully amplify *T. granarium* eDNA in a biosecurity context. Similarly, LAMP assays have also been developed to amplify DNA from microscopic pathogens and parasites in water samples and other fluid matrices (29–32), with one example of LAMP eDNA detection for a marine mollusc in *ex-situ* conditions (33). Between both molecular methods, qPCR has so far been used to successfully amplify insect eDNA from soil (17) and fecal (34) environmental matrices while LAMP-based assays had until this study not been tested to detect insect DNA in any environmental matrix, but failed to detect *T. granarium* eDNA in dust samples. Non-detection may be associated to the mechanism used in LAMP to amplify DNA, in which the six primers bind laterally to distinct sites using strand-displacement *Bst* DNA polymerase to amplify a single fragment of DNA (35). When used to target high quality DNA, LAMP has been shown to offer more timely detection of targeted species with higher sensitivity and accuracy than qPCR (35), however, the degraded and inhibited matrix in which *T. granarium* eDNA samples were collected may not offer suitable templates for all primers in the LAMP assay to amplify the target gene region. It is important to highlight that the assay was not developed for the purpose of eDNA-based testing and it is routinely used in Australia to confirm the identify of *T. granarium* specimens by targeting tissue-derived DNA (14). Future testing of TaqMan assays tested in this study for the purpose of eDNA-based detection will require more confirmed positive eDNA samples, however, the conditions in which this single sample was collected (i.e., private residence in Kambah: alive larva confirmed to be *T. granarium* in the sample) may be rare to find unless officers are actively looking to collect such samples.

Testing of both TaqMan assays using the Biomeme Franklin™ Thermocycler highlighted three important considerations that must be addressed for future field-based testing. Firstly, the technology can test only a small number of samples per day which may be a critical bottleneck during a biosecurity response. There are other portable technologies that could process a much greater number of samples per run in the field (e.g., Biomolecular systems Mic qPCR Cycler = 48 separate qPCR reactions), and larger numbers of samples could also be sent for laboratory-based testing if needed. However, further testing must consider the circumstances in which the use of portable technologies are most suitable. In this study, the Franklin™ Thermocycler and the Genie III were suitable to determine the identity of collected specimens by qPCR and LAMP, respectively, while qPCR-based assays were suitable to determine the presence of eDNA in a small number of samples. Secondly, officers require training to interpret eDNA-based testing results and determine appropriate contingencies in the event of false positives. False positive detections due to cross-contamination during collection in this study indicates that officers must readily assess when eDNA-based testing in the field would be unsuitable and sample collection unreliable. Field-based molecular testing would be another tool available to biosecurity officers to increase the likelihood of detection and to rapidly assess the presence/absence of a pest. However, detections would still require verification through further inspections and laboratory-based methods. Thirdly, deploying portable molecular technologies for pest detection in biosecurity requires the coordination of scientific, regulatory, and operational authorities to better determine the boundaries in which implementing this technology would ultimately be suitable. There is also an overarching need for an international collaborative framework aimed at unifying molecular sampling and analysis methodologies to facilitate the development of standards and encourage uptake of these techniques.

The operational use of laboratory and field-based eDNA molecular technologies requires standard operating procedures and legislation for implementation in biosecurity. Collection of eDNA in this study was subject to the priority of the biosecurity response, which was to clean and fumigate the contaminated area. For this reason, and in the absence of any policy on how and when to use eDNA-based methods, sampling in both responses was only undertaken after officers completed an initial examination at each site and proceeded to clean the area prior to fumigation as per biosecurity response requirements, aiming primarily to clean the area rather than collect samples for eDNA-based testing. The response in Fyshwick also highlighted the importance of using clean equipment to collect eDNA samples, as contamination of the vacuum cleaner that had been used in a previous biosecurity response with confirmed khapra beetle infestations was confirmed using the Franklin™ Thermocycler. As such, this study showed the critical need for standard protocols in sample collection and the need for controls in the eDNA workflow to indicate where potential sources of error are proliferating in biosecurity applications. In doing so, the results of this study were used to inform Australian Government Department of Agriculture, Water and the Environment, which funded further avenues of research to improve and develop standardized eDNA sampling methods for biosecurity applications. Ongoing research aims to detect *T. granarium* eDNA and environmental RNA in shipping containers to inform officers on the active presence of pest species. This research also aims to develop standard operating procedures to collect, store, extract and analyse environmental samples for the presence of pest species and implements rigorous collection methods to minimize sample cross-contamination within and between biosecurity responses.

In conclusion, this study shows that eDNA can be extracted and detected from dust samples collected as part of biosecurity border control and onshore responses in Australia. The extraction methods and TaqMan assays selected in this study were suitable for laboratory and field-based testing, however, both assays require further optimisation with confirmed positive eDNA samples to better define the sensitivity of each assay. The LAMP assay tested in this study failed to amplify *T. granarium* eDNA extracted from dust samples. The outcomes of this study show that sampling methods require the application of suitable protocols to ensure sample independence and avoid cross-contamination. The urgent conditions and circumstances under which biosecurity responses occur impose risks in field-based testing, as evidenced by false positive errors of both TaqMan assays due to cross-contamination. Environmental DNA testing could well complement current inspection methods in biosecurity responses by providing a tool to triage and prioritize efforts, however, there are still multiple obstacles that must be critically assessed before biosecurity officers could use portable molecular technologies as part of their biosecurity toolbox. Legislative, policy, and science-based guidelines that would determine how eDNA-based testing is to be undertaken in Australia are yet to be defined, however, this study provides an early view of how eDNA-based testing could greatly complement Australian biosecurity measures.

## Data Availability Statement

The datasets presented in this study can be found in online repositories. The names of the repository/repositories and accession number(s) can be found below: https://datadryad.org/stash, https://doi.org/10.5061/dryad.jwstqjq96.

## Ethics Statement

Written informed consent was obtained from the relevant individual(s) for the publication of any potentially identifiable images or data included in this article.

## Author Contributions

AT-G: conceptualization, data curation, writing—original draft preparation, and visualization. AT-G and DT: methodology. DT: software. AT-G and TW: sample collection. AT-G, KS, TW, and UD: formal analysis. DG, KS, TW, and UD: resources. DG and UD: project administration. AT-G, DG, and UD: funding acquisition. All authors: writing—review and editing. All authors contributed to the article and approved the submitted version.

## Funding

This study was funded by the Australian Government Department of Agriculture, Water and the Environment's Biosecurity Innovation Program (Project 182020) and supported by the Centre for Invasive Species Solutions.

## Conflict of Interest

The authors declare that the research was conducted in the absence of any commercial or financial relationships that could be construed as a potential conflict of interest.

## Publisher's Note

All claims expressed in this article are solely those of the authors and do not necessarily represent those of their affiliated organizations, or those of the publisher, the editors and the reviewers. Any product that may be evaluated in this article, or claim that may be made by its manufacturer, is not guaranteed or endorsed by the publisher.

## Supplementary Material

Supplementary 1 Integrated DNA Technologies gBlock gene fragments used as positive controls in TaqMan assays.

Supplementary 2 Assay optimization details for TaqMan assays tested in this study.
