# Supplementary material for: Detection of Khapra Beetle Environmental DNA Using Portable Technologies in Australian Biosecurity
Source: Front Insect Sci. 2022 Feb 11;2:795379. doi: 10.3389/finsc.2022.795379 (PMC10926498; doi:10.3389/finsc.2022.795379)
Supplement: Supplementary 1 — Integrated DNA Technologies gBlock gene fragments used as positive controls in TaqMan assays. [file Table_1.DOCX]

**Supplementary 1.** Integrated DNA Technologies gBlock gene fragments used as positive controls in TaqMan assays.

Black areas indicate primer binding regions, grey indicate probe binding regions and red areas indicate nucleotide inversions or poly-G inclusion to differentiate the synthetic oligo from real DNA fragments by sequencing. Artificial oligo composition and length for the Furui assay prevented doing sequence inversions.

| **Oligo** | **Targeted gene region** | **5'-sequence-3'** | **Product** |
| --- | --- | --- | --- |
| T. granarium Olson oligo | 16s | CTA GCC TGC TCC CTG ATT GAT TTT AAG AGC CGC AGT ATT TTG ACT GTG CGA AGG TAG CAT AAT AAT TAG TTT CTT TAT TGG GAA CTG GAA TGT CCA ACC ATT CGA AAT GAT CAC TGT CTC TTT TTT ATT TTG ATG AAT TTT ACT TTT GAG TTA AAA GGC TCA AAT TTT TTT AAA AGA CGA GAA GAC CCT ATA GAG TTT TAT TAT TTC TTT AAT TGT TAA ATT GTT AGT ATA GAA ATT TTC AAT TTT AGG TTA ATT TGG TTG GGG GGG | gBlocks® Gene Fragments 125-500 bp |
| T. granarium Furui oligo | ND6 NADH | CAA TTA AAA TCA GCC TTA TAT GAC TTC TCA TAC CAT TGA CAA CAC TCG CCA CCA TTT GCT TAAAGGGGGGTA TCA ATT CAT CAT TCC CAA CAT GAA ATC AAG AAT CAC TTC CAA TAG ACT TTA TCA CCC AA | gBlocks® Gene Fragments 125-500 bp |
