# Supplementary figures and images for: Detection of Khapra Beetle Environmental DNA Using Portable Technologies in Australian Biosecurity
Source: Front Insect Sci. 2022 Feb 11;2:795379. doi: 10.3389/finsc.2022.795379 (PMC10926498; doi:10.3389/finsc.2022.795379)

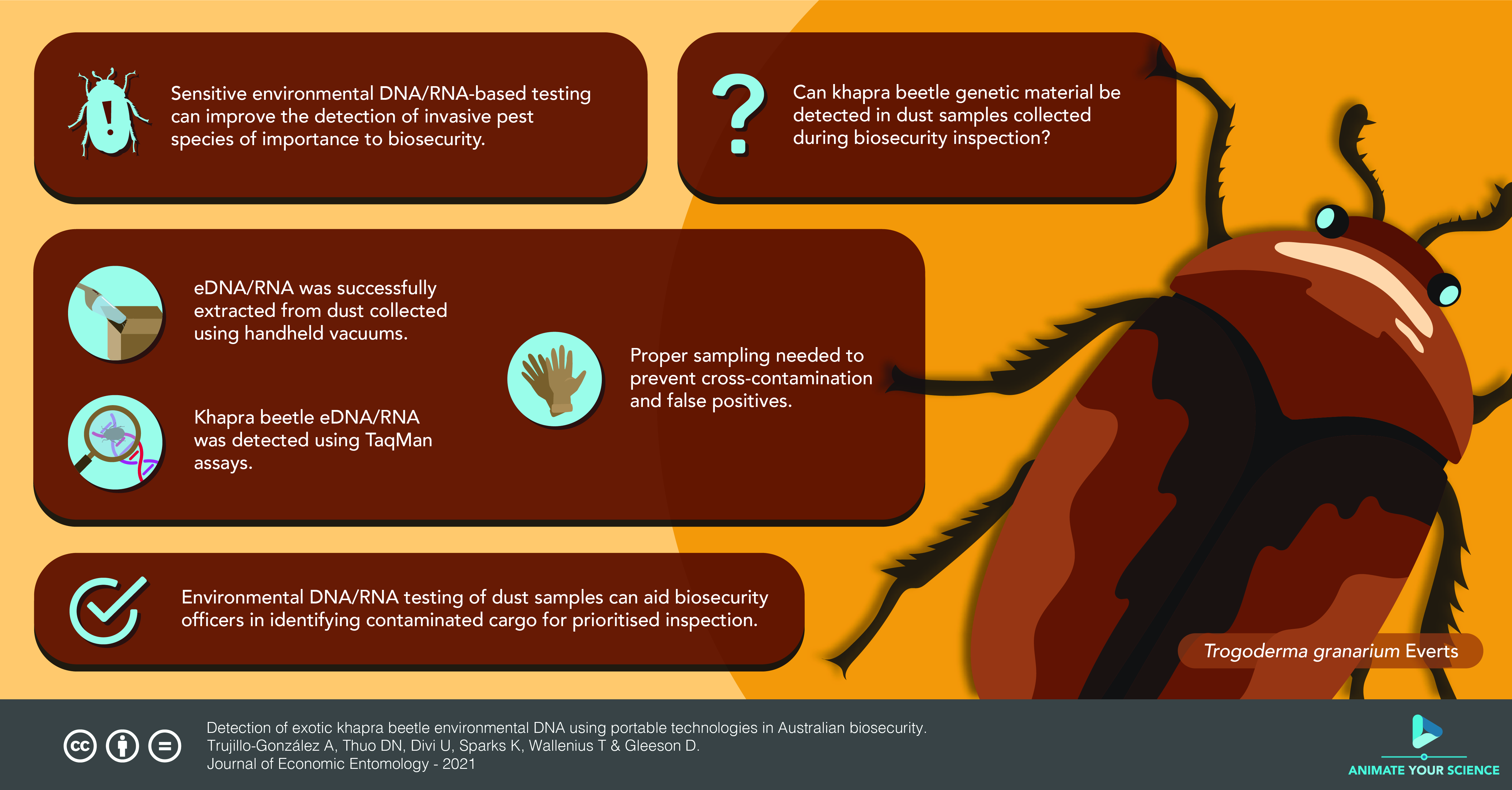

Supplement: Supplementary file 3 [file Image_1.JPEG]
